# Patient voice at the core of social prescribing: perspectives from people with lived experiences

**DOI:** 10.24095/hpcdp.44.6.09

**Published:** 2024-06

**Authors:** Sudi Barre

**Affiliations:** 1 Canadian Institute for Social Prescribing at the Canadian Red Cross, Mississauga, Ontario, Canada

**Keywords:** social prescribing, patient voice, person-centred practice, advocacy

**Highlights**

- I believe that creating space to listen deeply to patient voices and keeping this at the core of social prescribing practice is the key to successful social prescriptions.
- Social prescribing is a path of selfdiscovery, healing and empowerment that goes beyond traditional medical care.
- Social prescribing focusses on “what matters to you.”
- For social prescribing to truly be a person-centred practice, it is important that the voices of people with firsthand experiences continue to be a core part of the design, implementation and evaluation, as the number of social prescribing practices grow in Canada.

Dear Editors,

As a strong advocate for more equitable patient inclusion in research and the health care ecosystem, I believe that creating space to listen deeply to patient voices and keeping this at the core of social prescribing practice is the key to successful social prescriptions. 

Recently, I attended the third annual World Non-Communicable Disease (WNCD) conference, where I was given the opportunity to speak as a person who has personal experience with a noncommunicable disease. The majority of the attendees were doctors, researchers and academics. I was the only patient representative there. To me, social prescribing is a path of self-discovery, healing and empowerment that goes beyond traditional medical care. It makes space for what is critically important to me, which is to allow me and other patient populations to have a shared space in health care, rather than merely being treated as passive recipients of care. 

For social prescribing to truly be a person-centred practice that asks, “What matters to you?”, it is critical that our voice, the voice of people with firsthand experiences, continues to be a core part of the design, implementation and evaluation of the social prescribing initiative as the number of social prescribing practices grows in Canada.

I want to urge every social prescribing practitioner and champion to create space that encourages and enables patients to lead in their own care. If we do it in this way, as social prescribing becomes more prevalent and a solid treatment option, it is sure to offer all of us a better and more compassionate future. 

Sincerely,

Sudi Barre 

